# Individualized Brain Tissue Oxygen-Monitoring Probe Placement Helps to Guide Therapy and Optimizes Outcome in Neurocritical Care

**DOI:** 10.1007/s12028-020-01171-3

**Published:** 2020-12-16

**Authors:** Levin Häni, Mario D. Ropelato, Franca Wagner, Andreas Nowacki, Nicole Söll, Matthias Haenggi, Andreas Raabe, Werner J. Z’Graggen

**Affiliations:** 1 Department of Neurosurgery, Inselspital, Bern University Hospital, University of Bern, Freiburgstrasse, 3010 Bern, Switzerland; 2 Institute of Diagnostic and Interventional Neuroradiology, Inselspital, Bern University Hospital, University of Bern, Bern, Switzerland; 3 Department of Intensive Care Medicine, Inselspital, Bern University Hospital, University of Bern, Bern, Switzerland

**Keywords:** Traumatic brain injury, Brain hypoxia, Cerebral vasospasm, Subarachnoid hemorrhage

## Abstract

**Background/Objective:**

In order to monitor tissue oxygenation in patients with acute neurological disorders, probes for measurement of brain tissue oxygen tension (ptO_2_) are often placed non-specifically in a right frontal lobe location. To improve the value of ptO_2_ monitoring, placement of the probe into a specific area of interest is desirable. We present a technique using CT-guidance to place the ptO_2_ probe in a particular area of interest based on the individual patient’s pathology.

**Methods:**

In this retrospective cohort study, we analyzed imaging and clinical data from all patients who underwent CT-guided ptO_2_ probe placement at our institution between October 2017 and April 2019. Primary endpoint was successful placement of the probe in a particular area of interest rated by two independent reviewers. Secondary outcomes were complications from probe insertion, clinical consequences from ptO_2_ measurements, clinical outcome according to the modified Rankin Scale (mRS) as well as development of ischemia on follow-up imaging. A historical control group was selected from patients who underwent conventional ptO_2_ probe placement between January 2010 and October 2017.

**Results:**

Eleven patients had 16 CT-guided probes inserted. In 15 (93.75%) probes, both raters agreed on the correct placement in the area of interest. Each probe triggered on average 0.48 diagnostic or therapeutic adjustments per day. Only one infarction within the vascular territory of a probe was found on follow-up imaging. Eight out of eleven patients (72.73%) reached a good outcome (mRS ≤ 3). In comparison, conventionally placed probes triggered less diagnostic and therapeutic adjustment per day (*p* = 0.007). Outcome was worse in the control group (*p* = 0.024).

**Conclusion:**

CT-guided probe insertion is a reliable and easy technique to place a ptO_2_ probe in a particular area of interest in patients with potentially reduced cerebral oxygen supply. By adjusting treatment aggressively according to this individualized monitoring data, clinical outcome may improve.

## Introduction

Cerebral oxygen supply is a critical parameter in the treatment of patients with acute cerebral disorders such as traumatic brain injury (TBI) and subarachnoid hemorrhage (SAH). Cerebral tissue hypoxia poses cellular metabolism and viability at risk and can cause secondary brain injury in these patients. Invasive measurement of brain tissue oxygen tension (ptO_2_) allows continuous monitoring of cerebral oxygenation and early detection of evolving ischemia [1–5]. ptO_2_ measurements are taken by introducing a small, oxygen-sensitive catheter into the brain tissue through a burr hole. A drop of ptO_2_ below 10 mmHg is associated with cerebral ischemia and cell death [6, 7]. Accordingly, lower ptO_2_ values are associated with unfavorable outcome and higher mortality in clinical studies of patients with TBI [8, 9]. Importantly, tissue hypoxia can occur even in the absence of macrovascular ischemia or intracranial hypertension; thus, ptO_2_ measurements provide additional prognostic information [10–12]. Several retrospective studies as well as a recent, prospective randomized phase II trial suggest a clinical benefit with improved outcome and lower mortality when guiding therapy according to ptO_2_ and intracranial pressure (ICP) compared to ICP alone in patients suffering from TBI [13–19]. Currently, the randomized controlled phase III BONANZA trial (Australian New Zealand Clinical Trials Registry ACTRN12619001328167) as well as the BOOST-3 trial (clinicaltrials.gov NCT03754114) is recruiting patients with severe TBI in order to provide further evidence. In aneurysmal SAH, impaired ptO_2_ values were shown to correlate significantly with angiographic intracranial arterial caliber reduction, cerebral infarction, and survival [12, 20–22]. Furthermore, ptO_2_ directed therapy was shown to be associated with improved outcome after SAH [23]. However, the association of lower ptO_2_ values and outcome was only weak in clinical studies of patients suffering from SAH and results have been conflicting [12, 24, 25]. Importantly, the ptO_2_ measurement is focal and covers only a very limited brain volume in the range of a few cubic millimeters [2, 4]. Thus, detection of critical cerebral perfusion due to vasospasms in SAH patients depends on the positioning of the ptO_2_ probe, which might explain the conflicting results [25, 26].

For routine clinical use, a ptO_2_ probe is often inserted through a standard frontal burr hole and placed in the white matter of the right frontal lobe. Many institutions use triple or quadruple lumen bolts for multimodal intracranial monitoring consisting of ICP, ptO_2_, cerebral blood flow, microdialysis, brain temperature, and/or intracranial electroencephalography [27]. While this approach minimizes trauma by limiting the number of burr holes, it does not take into account the individual patient’s pathology. The brain metabolic profile largely depends on the probe location with respect to a brain lesion [28, 29]. Hence, selective placement of the probe into a particular area of interest is crucial to provide clinically useful information [1]. Hence, non-specific placement of the probe will lower the diagnostic value of the measurement and ultimately jeopardize the outcome of the patient.

Here, we describe a new, fast, and easy to apply technique using CT-guidance to place the ptO_2_ probe in a particular area of interest, which is selected for each patient individually. The aim of the present study was to assess the accuracy, applicability, and clinical value of CT-guided ptO_2_ probe placement. We hypothesized that by CT-guidance, we are able to place the ptO_2_ probes in a particular area of interest with a high rate of success, which would translate into improved monitoring and care of patients with acute neurological disorders with the potential to improve outcome.

## Methods

### Standard Protocol Approvals, Registrations, and Patient Consents

This is a retrospective cohort study of patients treated at the University Hospital of Bern. We obtained approval from the local ethics committee (Kantonale Ethikkommission Bern, Switzerland) for this study (Project ID 2019-00921), which, because of the retrospective analysis of routine data, waived the need for individual informed consent for the study and allowed the further use of health care data if the patient or next-of-kin had followed the general consent procedure.

### Patient Population

We included all patients, who had a ptO_2_ probe inserted between January 2010 and April 2019. Inclusion criteria for the study population were insertion of a ptO_2_ probe by CT-guidance, which was routinely performed between October 2017 and April 2019, and age ≥ 18 years. For the historical control group, inclusion criteria were conventional, free-hand insertion of a ptO_2_ probe, which was routine before October 2017, and age ≥ 18 years. Inclusion was irrespective of the underlying pathology, but most patients suffered either from TBI or SAH.

### Intervention

The indications for placement of a ptO_2_ probe were:

1. Risk of evolving ischemia because of cerebral vasospasms and inability to monitor clinically in patients suffering from aneurysmal SAH.
2. Critical ICP elevation despite maximal conservative therapy and potential need for intermittent moderate hyperventilation in patients suffering from traumatic brain injury or intracerebral hemorrhage.

We determined for each patient individually an area of interest for ptO_2_ measurements based on clinical grounds as well as radiological signs of hypoperfusion on CT-perfusion imaging, vasospasm of the basal cerebral arteries on angiography, or perifocal edema of mass occupying lesions. Generally, we aimed at placing the ptO_2_ probe in an area at risk for hypoxic brain injury. In patients with vasospasms in the territories of the anterior cerebral artery (ACA) and the middle cerebral artery (MCA), we aimed for the watershed zone between these two supply areas. Contrary, in patients with vasospasms in only one of these vessels, we aimed for the specific supply area. If the patient had a preexisting intraparenchymal hematoma, contusion, or infarction, that area was avoided. In patients with traumatic brain injury, we aimed for the frontal lobe on the side, which was more affected by intracranial injuries or for the right frontal lobe, if both sides were equally affected. A target area was chosen at a safe distance from hemorrhagic contusions and preexisting external ventricular drains. Anatomically, the probes were aimed for the watershed zone between the ACA and MCA supply area or in the anterior MCA territory if access to the watershed zone was restricted by a preexisting external ventricular drain.

The technique of CT-guided ptO_2_ probe insertion was modified and derived from our recent description of CT-guided insertion of external ventricular drains [30]. The exclusive use of single lumen bolts allowed insertion of ptO_2_ probes irrespective of the location other neuro-monitoring devices. Because the commercially available bolt-kit ptO_2_ probe (LICOX, Integra LifeSciences, Plainsboro, NJ) has to be inserted at a fixed depth of 30 mm from the inner table or 35 mm from the outer table of the skull bone into the brain, we projected a sphere with a radius of 35 mm with the area of interest in the center of the sphere onto the patient’s CT scan. We then selected a point on the intersection line with the outer table of the skull, which was suitable for burr hole placement and probe insertion. The distance to well-defined craniometrics landmarks (midline, nasion, bregma, coronal suture, or an existing external ventricular drain) was measured on the CT scan. We transferred these measurements onto the patient’s skull and placed the burr hole accordingly. Burr hole placement and bolt insertion were performed in a standard manner in the CT or angiography suite. We use povidone-iodine (Betadine; Mundipharma, Basel, Switzerland) or chlorhexidine (chlorhexidine 2% alcoholic uncolored, B. Braun Medical, Melsungen, Germany) and surgical drapes for skin preparation. The surgeon incised the skin over approximately 5 mm at the site of the planned burr hole. The burr hole was drilled with a manually operated twist drill. After the dura was perforated, we screwed the bolt of the ptO_2_ probe (LICOX, Integra LifeSciences, Plainsboro, NJ) superficially into the skull in the approximate angle necessary to reach the area of interest. We then performed an immediate bolt CT scan to verify the trajectory of the bolt. In order to reduce the radiation dose, the scan covered only the supraorbital part of the skull including the bolt end down to roof of the third ventricle. The CT data set was immediately reconstructed along the trajectory of the bolt. If necessary, we adjusted the trajectory by adapting the angulation of the bolt. In this case, a second bolt CT was performed to confirm the correct trajectory. This procedure was repeated until the correct bolt angulation was found. Then, the surgeon screwed the bolt firmly into the skull and inserted the sheath of the ptO_2_ probe. Another CT scan was acquired to confirm the correct location of the sheath. After insertion of the ptO_2_ probe, a final postoperative CT scan was performed to assess the correct location of the tip of the ptO_2_ probe in the area of interest.

If duration of ptO_2_ monitoring exceeded one week, we routinely exchanged probes.

**Fig. 1:**
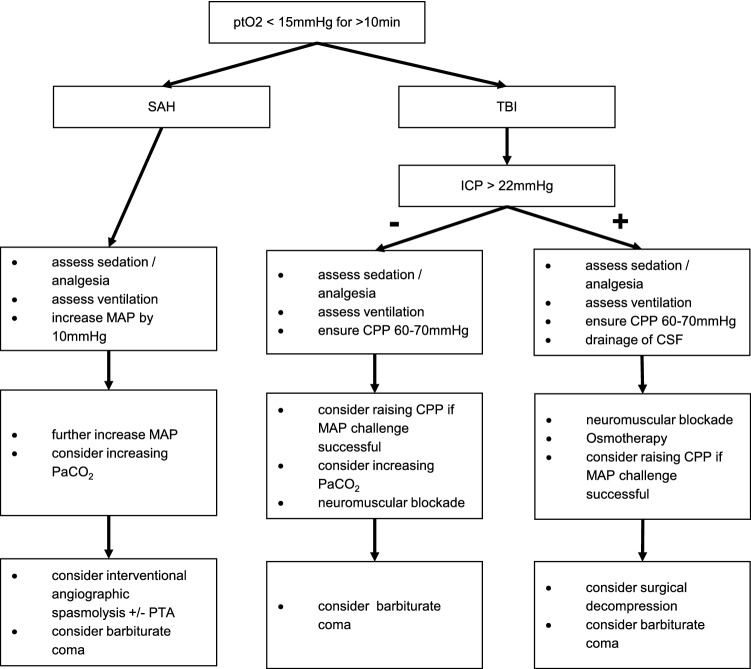
Therapeutic algorithm in case of decline of ptO_2_ measurements. *CPP *cerebral perfusion pressure; *CSF* cerebrospinal fluid; *MAP* mean arterial pressure; *PTA* percutaneous transluminal angioplasty; *SAH* subarachnoid hemorrhage; *TBI *traumatic brain injury

Our threshold for the initiation of diagnostic or therapeutic measures is a ptO_2_ measurement ≤ 15 mmHg for ≥ 10 min. In cases with an evident reason for ptO_2_ decline, e.g., documented vasospasms after SAH, no further diagnostic studies were initiated, but therapeutic measures were taken directly. These consisted of medical measures first (e.g., adjustment of mean arterial pressure in case of vasospasm related misery perfusion or medical therapy for increased ICP) and in refractory cases, interventional measures such as local spasmolysis or angioplasty, or surgical interventions (e.g., hemicraniectomy). A detailed description of the therapeutic algorithm is given in Fig. 1. The algorithm is consistent with the BONANZA algorithm and in large parts with the BOOST-II algorithm, although inspiratory oxygen fraction was not increased to correct ptO_2_ values [16].

### Data Analysis

The primary endpoint was the successful CT-guided positioning of the ptO_2_ probe inside the area of interest in the study group. The correct location was evaluated by two raters (L. H. and M. D. R.). Based on the target area specified in the operation report, as well as the consideration of clinical and imaging characteristics of each individual patient, each rater retrospectively placed a sphere with a radius of 1 cm (volume of 4.2 ml) with the point of major interest in the center onto the preinterventional CT scan. Overlap with preexisting infarcts, hematomas, and contusions was avoided. These spheres represented the target location. We fused the pre- and postinterventional images then and evaluated the location of the probe with respect to the sphere. If the tip of the probe was inside the sphere, we considered the placement correct. Conversely, if the tip of the probe was located outside the sphere, we considered the placement incorrect. If both raters agreed on the correct placement of the probe, the primary endpoint was reached and the positioning successful. If one of the raters considered the tip inside the target location, while the other rater did not, or if both raters agreed on the incorrect placement, we considered the placement unsuccessful and the primary endpoint was not reached. For image manipulation and fusion, the software Elements (Brainlab, Munich, Germany) was used.

Secondary endpoints included radiological and clinical parameters. We evaluated for the study and the historical control group complications from probe insertion, clinical consequences from ptO_2_ measurements, as well as clinical outcome according to the modified Rankin Scale (mRS) after 6 months. A good outcome was considered a mRS score of ≤ 3.

Radiological outcome was assessed by a board-certified, senior neuroradiologist (F. W.) and included complications from probe insertion, determination of the vascular territory of the probe, and assessment of vasospasm, hypoperfusion, or ischemia over the course of ptO_2_ monitoring until 7 days thereafter inside or outside the target area of the probe. Furthermore, we recorded the appearance of any new parenchymal lesions or ischemia on the latest imaging study available from each patient.

Clinical data and ptO_2_ values were extracted from the institutional electronic Patient Data Management System (Centricity™ Critical Care, General Electric Company, GE Healthcare, USA). The system automatically documents all ptO_2_ and ICP values, hemodynamic, and respiratory variables in intervals of 2 min. Further clinical data such as GCS score, fluid balances, and administered drugs are entered manually by the bedside team.

Additionally, for the study group only, the total number of CT scans for probe insertion as well as the cumulative radiation exposure was documented for each patient. The cumulative radiation dose was calculated as the sum of the dose-length product (DLP) of each CT scan:

$$ {\text{DLP }} = {\text{ CTDI}}_{{{\text{Vol}}}} {\text{x nT}}, $$

where CTDI = computed tomography dose index and nT = product of the number of slices and slice thickness. To calculate the effective dose (E), a conversion factor of 0.0021 was applied:[31]

$$ {\text{E }} = {\text{ DLP x }}0.00{21}. $$

The duration of the intervention was derivated from the acquisition time of imaging data. The total duration was defined as the time interval between the last diagnostic scan, i.e., before insertion of the bolt, and the final confirmatory scan. Thus, the total duration includes the time for diagnostic imaging analysis, target selection, entry point calculation, bolt insertion, analysis and correction of the trajectory, any additional scans, and insertion of the probe. The extra duration of using CT-guidance was defined as the time interval between the first scan with the bolt in situ and the final confirmatory scan. The extra duration represents the time for the analysis and correction of the trajectory, any additional scans, and insertion of the probe.

Based on the three vascular territories available (ACA, watershed zone, and anterior MCA), we grouped the correctly placed probes together and measured the location of the cranial entry points and trajectories of the ptO_2_ probes. The location of the entry point as distance from the midline in the coronal plane and as distance from the coronal suture in the sagittal plane was measured. Furthermore, the probe trajectory was assessed in degrees angulation in a sagittal projection with reference to the Frankfurt horizontal plane as well as in a coronal projection with reference to the midline.

### Statistics

We will report results of the primary outcome descriptively. In case a patient had bilateral probes placed, we evaluated both sides independently.

For comparison of entry sites and trajectories between groups of different vascular territories, a Kruskal–Wallis test was applied. Continuous variables are reported as mean and standard deviation. We compared the study group and the control group using a Mann–Whitney U test for continuous variables and Chi-Square or Fisher’s exact test for nominal variables. Statistical analysis was performed using the statistical software SPSS (IBM, Version 25).

We addressed missing values first by re-analyzing the source data or, in case no value was retrievable, pairwise deletion.

## Results

### Patient Population

Twelve patients had a CT-guided ptO_2_ probe placed between October 2017 and April 2019. One patient had to be excluded because of his age < 18 years. Six of them had a unilateral probe placed, while the other five underwent bilateral probe placement. Thus, 16 probes in eleven patients were available for analysis in the study group (Table 1).

**Table 1:** Clinical and radiological details of included patients

Patient	Condition	Sex	Age (years)	Side	Probe in target	Duration of measurement (days)	Infarcts on last imaging FU^§^	Diagnostic studies^π^	Therapeutic measures^ω^		mRS at 6 months
									Noninvasive	Invasive	
1	SAH	m	34.88	R + L	y/y	12 (R)/10 (L)	Out (R)/in (L)	2 (R)/1 (L)	8 (R)/6 (L)	7 (R)/2 (L)	4
2	SAH	f	63.02	L	y	8	Out	0	3	4	1
3	SAH	f	56.09	R + L	n (R)/y (L)	12 (R + L)	None/out (L)	1 (R)/2 (L)	1 (R)/4 (L)	2 (R)/4 (L)	2
4	SAH	f	56.93	R	y	2	Out	0	0	0	1
5	TBI	f	32.44	R	y	7	None	0	0	0	2
6	TBI	m	33.90	L	y	1	None	0	0	0	1
7	SAH	f	66.60	R + L	y/y	12 (R)/11 (L)	None (L + R)	1 (R)/1 (L)	4 (R)/2 (L)	1 (R)/0 (L)	1
8	SAH	f	56.44	L	y	6	None	0	0	0	4
9	RCVS	m	44.50	R + L	y/y	6 (L + R)	Out (R + L)	1 (R)/0 (L)	1 (R)/1 (L)	0	4
10	ICH	f	23.79	L	y	10	In	0	4	2	1
11	SAH	f	46.93	R + L	y/y	13 (L + R)	None (R + L)	0	0 (R)/2 (L)	0 (R)/1 (L)	1

^§^ = presence of ischemic infarcts on the last available imaging follow-up, either inside (in) or outside (out) of the area of the probe placed; ^π^ = number of diagnostic studies initiated by declining ptO_2_ measurements; ^ω^ = number of therapeutic measures initiated by declining ptO_2_ measurements

*f* Female, *FU* Follow-up, *ICH* Intracerebral hemorrhage, *L* Left side, *m* Male, *mRS* Modified Rankin Scale, *n* No, *n/a* Not applicable, *R* Right side, *RCVS* Reversible cerebral vasoconstriction syndrome, *SAH* Subarachnoid hemorrhage, *TBI* Traumatic brain injury, *y* Yes

Twenty-one patients had a ptO_2_ probe placed in conventional, free-hand technique between January 2010 and October 2017. From this group, we excluded six patients because of withdrawal of care within 24 h of probe placement (*n* = 3), lack of meaningful ptO_2_ values due to a technical error (*n* = 1), or missing documentation (*n* = 2). In the remaining 15 patients of the control group, eleven patients had bilateral and four patients unilateral probes placed. Thus, 26 probes were analyzed in the control group. Baseline characteristics of study and control patients did not differ significantly (Table 2). However, there was a trend toward younger age in the study group.

**Table 2:** Comparison of baseline characteristics between study group (CT-guided placement) and control group (conventional placement)

	Study group *n* = 11	Control group *n* = 16	*p* value
Age (years)	46.87 (± 14.09)	57.61 (± 13.90)	0.077
Female sex	8 (72.7%)	9 (60.0%)	0.683
Bilateral placement	5 (45.5%)	11 (73.3%)	0.228
Disease			0.628
TBI	2 (18.2%)	2 (13.3%)	
SAH	7 (63.6%)	12 (80%)	
ICH	1 (9.1%)	1 (6.7%)	
other	1 (9.1%)	0	

Mean values with standard deviation are given for continuous variables

*ICH* Intracerebral hemorrhage, *SAH* Aneurysmal subarachnoid hemorrhage, *TBI* Traumatic brain injury

Mean duration of ptO_2_ monitoring was longer in the study population (8.81 days, range 1–13 days) than in the control group (4.33 days, range 1–8 days) (*p* < 0.001). Clinical and radiological follow-up data were available for all patients of the study group, whereas no radiological follow-up was available for three patients with four probes of the control group.

### Probe Placement in the Study Group

The primary endpoint of successful probe placement was met with 15 (93.75%) probes in the study group. Rater 1 and 2 considered 15 probes being inside the target area, while both raters agreed on the misplacement of the same probe (right sided probe of patient 3).

Radiologically, two probes were inserted into the vascular territory of the ACA, three probes into the watershed zone, and 11 probes into the anterior part of the vascular territory of the MCA. Perfusion imaging at time of probe insertion revealed hypoperfusion in the territory of ten probes. For five probes, perfusion imaging showed normal perfusion and for one probe, no perfusion imaging was available at insertion. Of note, no patient showed hypoperfusion exquisitely outside the vascular territory of the probe.

We placed ten probes under guidance of our CT scanner (128-slice CT scanner, Somatom Definition Edge, Siemens Healthcare, Erlangen, Germany) and six probes in the angiography suite with guidance of a Flat-Panel Detector Computed Tomography (FD-CT, DYNA-CT; Siemens “Artis” Models, Siemens Healthcare, Erlangen, Germany).

**Fig. 2:**
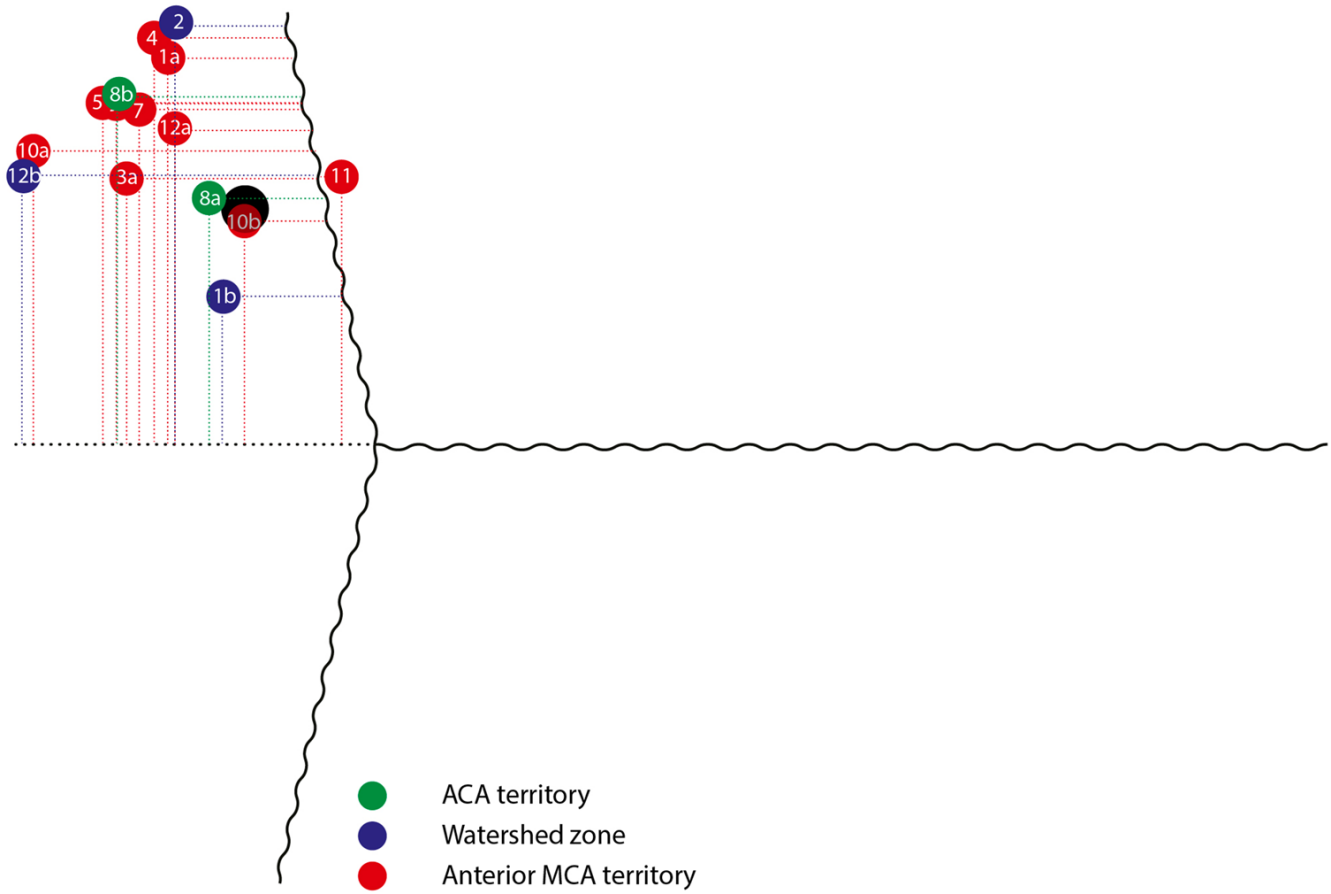
Individual entry points of all successfully placed probes with relation to the midline (horizontal line) and coronal suture (vertical line). All entry points are projected on the same side of the skull for simplification. Entry points of probes in the ACA territory are depicted green, while those of probes in the watershed zone are blue and the anterior MCA territory red. While most entry points cluster 3.5–4 cm lateral to the midline and 2 cm anterior to the coronal suture (to the left in the image), the different groups of vascular territories did not diverge significantly. The larger, black dot marks Kocher’s point as an illustrative reference

**Fig. 3:**
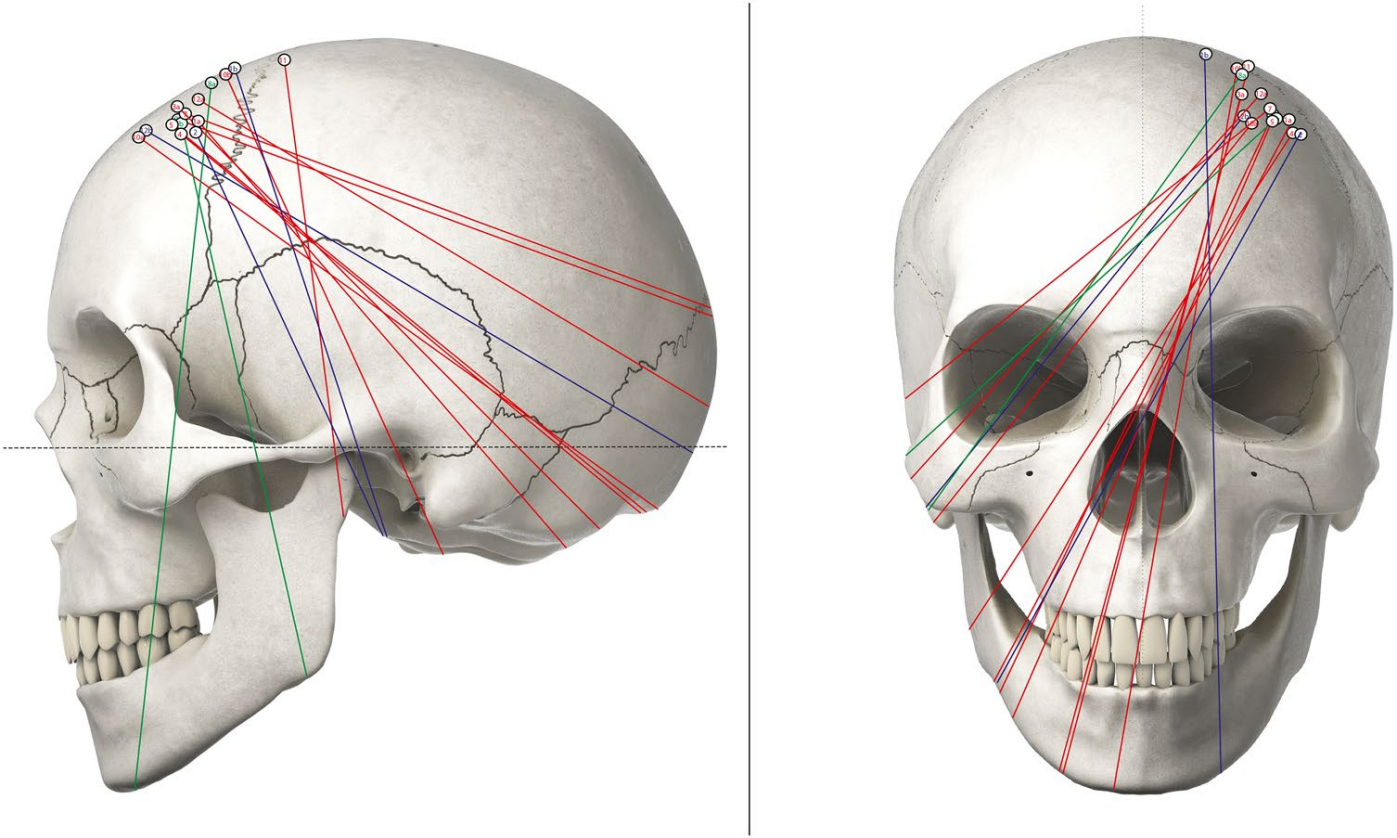
Individual trajectories of all successfully placed probes with relation to the axial plane parallel to the Frankfurt horizontal plane (left) and the sagittal plane in the midline (right). All trajectories are projected on the same side of the skull for simplification. Trajectories of probes in the ACA territory are depicted green, while those of probes in the watershed zone are blue and the anterior MCA territory red. As a rule of thumb, the insertion into the anterior MCA territory was angulated aiming at the nasion in the coronal and the inion in the sagittal plane. Contrary, the insertion into the ACA territory was angulated at the contralateral superior temporal line in the coronal plane and the zygomatic protuberance in the sagittal plane. For the watershed zone, an angulation aiming at the nasion in the coronal plane and the tragus in the sagittal plane serves as a rough estimate

The location of the cranial entry points and the trajectories of the ptO_2_ probes for different vascular territories are given in Table 3. Individual entry points and trajectories are displayed in Figs. 2 and 3. Of note, the choice of an entry point was limited due to an existing external ventricular drain on the same side in 8/16 (50%) of the probes placed. While comparison of entry points showed no significant differences for different vascular territories (*p* = 0.934 for distance to midline, *p* = 0.922 for distance to coronal suture), a nonsignificant trend for different angulations was found in the sagittal projection (*p* = 0.107).

**Table 3:** Insertion site and trajectory of ptO_2_ probes according to vascular territory

	ACA	Watershed	MCA	*p* value*
Midline (cm)	3.79 (± 0.92)	3.55 (± 1.73)	4.05 (± 0.72)	0.934
Coronal suture (cm)	1.93 (± 0.60)	2.24 (± 1.30)	1.90 (± 1.03)	0.922
Sagittal (degrees)	86.70 (± 13.44)	55.87 (± 22.24)	42.89 (± 19.39)	0.107
Coronal (degrees)	41.70 (± 8.49)	22.23 (± 21.00)	28.15 (± 13.37)	0.392

Location of the cranial entry point and trajectory of the ptO_2_ probes for different vascular territories. Mean± standard deviation is given for all values. The location of the entry points is given as distance from the midline in a coronal plane as well as distance from the coronal suture in the sagittal plane. The trajectories are given in degrees angulation in a sagittal projection with reference to the Frankfurt horizontal plane as well as in a coronal projection with reference to the midline

*ACA* Anterior cerebral artery territory, *Watershed* Watershed zone between the anterior and middle cerebral artery territory, *MCA* Anterior portion of the middle cerebral artery territory, *cm* Centimeter.* Kruskal–Wallis test

The average number of CT scans performed for probe placement per patient was 3.91 (± 2.07). The mean DLP and effective dose were 1595.17 (± 769.00) mGycm and 3.34 (± 1.61) mSv, respectively, per patient.

We repositioned the bolt in order to adjust the trajectory before insertion of the probe sheath with the stylet on average 0.8 times (range 0–4). The average total duration of probe placement was 40 min 40 sec (± 20 min 43 sec). The average extra duration for CT-guided probe placement was 15 min 28 sec (± 9 min 45 sec).

### Therapeutic Implications of ptO_2_ Measurement

In the study group, declining ptO_2_ measurements triggered 68 diagnostic or therapeutic interventions as a direct consequence of ptO_2_ monitoring. With a total duration of 141 days of measurement, this results in a rate of 0.48 interventions per probe per day of measurement. Considering a mean duration of 8.81 days of monitoring per probe, a unilateral ptO_2_ probe triggered on average 4.23 diagnostic or therapeutic adjustments per patient. Out of 68 diagnostic or therapeutic interventions, 9 consisted of diagnostic cranial imaging, while 59 interventions were therapeutic adjustments. Thirty-six were noninvasive therapeutic adjustments (0.25 per probe per day). The majority of them included increasing mean arterial pressure to optimize cerebral perfusion, optimizing respiratory parameters, increasing sedation, or medical therapy to decrease ICP. Additionally, ptO_2_ measurements initiated 23 invasive therapeutic interventions (0.16 per probe per day). All of them consisted of angiographic interventions (spasmolysis or angioplasties).

In comparison, the average rate of diagnostic and therapeutic interventions per probe per day triggered by declining ptO_2_ measurements was significantly less frequent in the control group (0.15 diagnostic or therapeutic interventions per probe per day of measurement, *p* = 0.007). The rate of noninvasive and invasive therapeutic adjustments per probe per day was also significantly less frequent in the control group (0.07 and 0.04, respectively; *p* = 0.002 and 0.011, respectively). Considering a mean duration of 4.33 days of measurement, a unilateral probe triggered in the control group on average 0.65 diagnostic or therapeutic adjustments.

### Clinical and Radiological Outcome

**Fig. 4:**
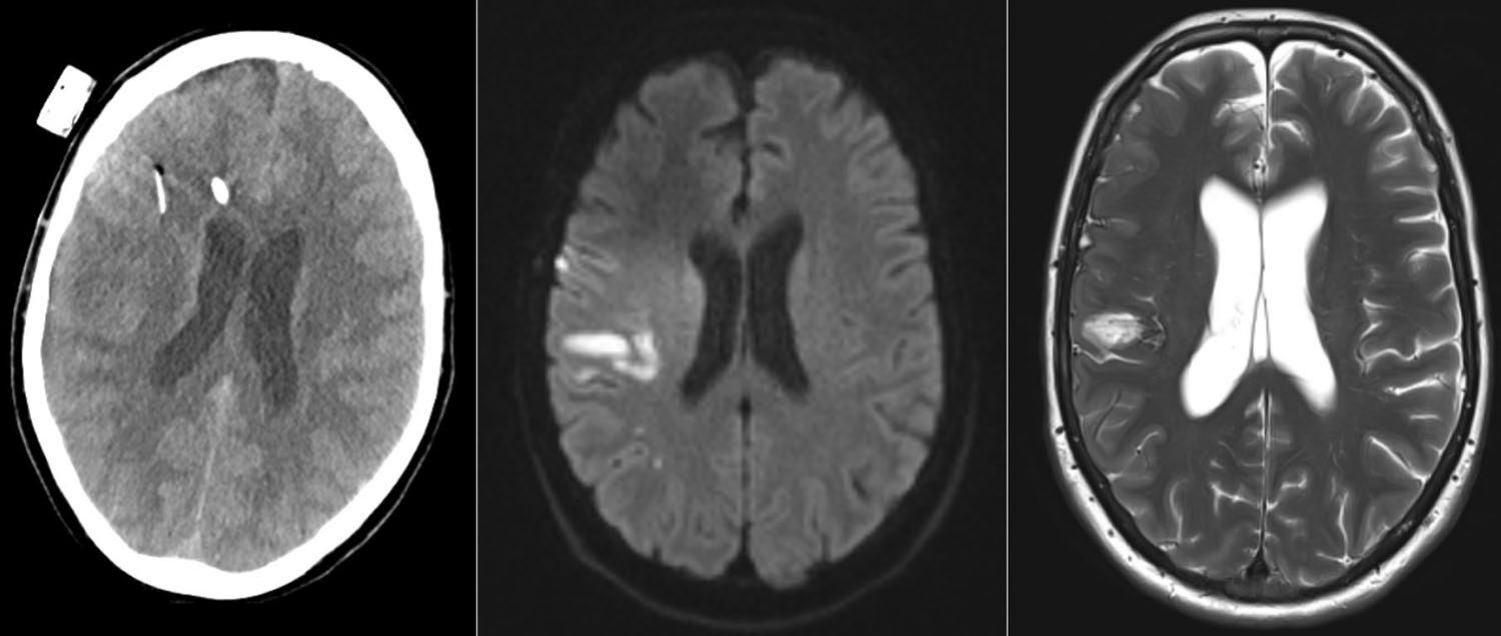
Infarction outside the vascular territory of the probe in patient 4. The patient suffered from aneurysmal subarachnoid hemorrhage with subsequent severe vasospasms causing an ischemic infarction. While the probe was inserted into the anterior MCA territory (left, brain CT without contrast), an ischemic infarction developed in the central MCA territory and was visible on initial MR scan (middle, diffusion-weighted imaging) and on follow-up MR imaging (right, T2-weighted image)

**Fig. 5:**
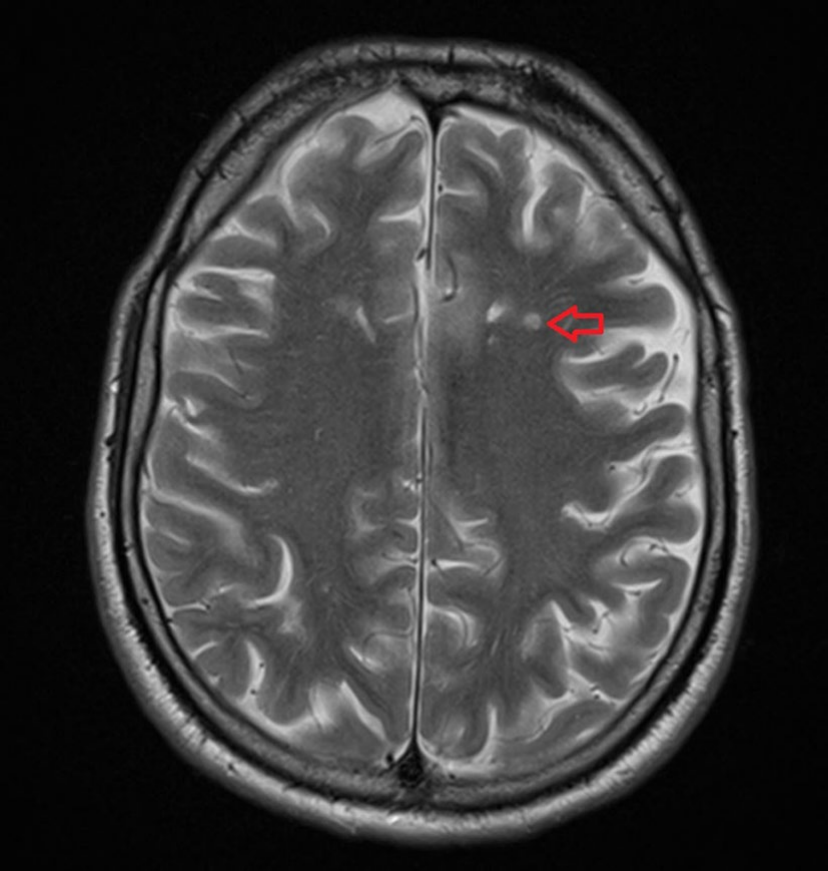
The only ischemic lesion inside the vascular territory of the probe occurred in the left frontal lobe of patient 1 (T2-weighted image of brain MR scan). The patient suffered from aneurysmal subarachnoid hemorrhage with subsequent severe vasospasm. The lesion resembles a small demarcated lacunar infarct (arrow). The most likely cause of ischemia in this patient was either vasospasm or an embolic lesion secondary to an angiographic intervention. However, it remains unclear, whether it was related to the introduction of the probe itself or represents a true ischemic lesion

The mean duration of radiological follow-up in the study group was 5.58 months (range 1–14). MRI was available in five and a CT scan in six patients. Radiological long-term follow-up showed no ischemic infarcts in eight hemispheres with a probe. A proximal infarct, inside the vascular supply territory captured by the probe, was found in one hemisphere. Distal infarcts, outside the vascular territory captured by the respective probe, were present in six hemispheres. The most frequent location of an infarct outside the area captured by the probe was the central MCA territory with a probe placed in the anterior MCA territory or the watershed zone (Fig. 4). In the patient with an infarct inside the vascular territory of the probe, the anterior MCA territory was affected. The lesion in the territory of the probe resembles a small lacunar ischemia (Fig. 5). In that patient, eight therapeutic adjustments were made based on the ptO_2_ measurement in the respective hemisphere. Out of four patients, who had no diagnostic or therapeutic measures initiated based on the ptO_2_ measurements, three had no ischemic infarcts at all on the last follow-up imaging, while one had an ischemic infarct outside the vascular territory of the probe.

At 6 months, 8 patients (72.73%) of the study group showed a good outcome (mRS ≤ 3), whereas 3 patients showed a mRS of 4. Of note, 6 patients reached an excellent outcome with a mRS of 0 or 1. Comparing the distribution of mRS scores yielded a significantly better outcome in the study group compared to the control group (*p* = 0.024). While 72.73% of patients in the study group reached a good outcome, only 46.67% in the control group did so (*p* = 0.246). Mortality in the study group was significantly lower than in the control group (0% vs. 40%; *p* = 0.024).

### Complications

None of the patients of the study group suffered from a direct complication of probe placement. While a small epi- or subdural hematoma was visible on the postoperative CT scan in five probes, none of them was clinically relevant or required any intervention. Median diameter of the hematoma measured on coronal images was 4.0 mm (range 2.9–6.2 mm). In one patient, a new intracerebral hematoma was visible on follow-up scans, which was most likely related to the removal of the probe. The patient remained asymptomatic for this bleeding. One patient of the study group and two patients in the control group developed ventriculitis, which were probably related to concomitant placement of an external ventricular drain. No other infectious complications occurred.

## Discussion

### Probe Placement

Our results demonstrate the feasibility of accurate, individualized placement of ptO_2_ probes by CT-guidance. By using this easy technique, we placed the probes in the area of interest with a success rate of 93.75%. Probe misplacement occurred due to procedural errors and anatomical difficulties. Procedural errors comprise failure to align the imaging reconstruction of the trajectory perfectly with the bolt and incorrect measurement of the distance along the trajectory. Furthermore, insertion in a flat angle in relation to the skull can be difficult, because drilling in a flat angle is technically more challenging and the insertion of the bolt through a longer bony tunnel restricts the number and extent of possible corrections of the trajectory. In our study, we did not perform further correctional maneuvers once the probe was inserted.

Furthermore, our analysis highlights the importance of the trajectory chosen rather than the entry point in order to reach a specific vascular territory. Probes were usually inserted 3.5–4 cm lateral to the midline and approximately 2 cm in front of the coronal suture. In case of an existing external ventricular drain with the entry site at the Kocher’s point, the insertion site of the ptO_2_ probe can be estimated 1–2 cm further anterior and lateral to the existing drain. This distance should not be less than 1 cm–avoid conflicts with the bolt of the external ventricular drain.

As a rule of thumb, the insertion into the anterior MCA territory needs an angulation aiming at the nasion in the coronal and the inion in the sagittal plane. The insertion into the ACA territory warrants a steep trajectory aiming at the zygomatic protuberance in the sagittal plane and at the contralateral eyeball in the coronal plane. For the watershed zone, an angulation aiming at the nasion in the coronal plane and the tragus in the sagittal plane serves as a rough estimate. Obviously, the angulation to the coronal plane should be increased the more lateral the entry point is chosen.

The total duration for ptO_2_ probe placement including the time for diagnostic imaging analysis, target selection, entry point calculation, bolt insertion, analysis and correction of the trajectory, any additional scans, and insertion of the probe was 40 min 40 sec. This burdens institutional resources, since the CT scanner is occupied for this duration. However, the extra duration of CT-guided placement compared to conventional free-hand placement was only 15 min 28 s. In our study, no patient was transferred to the CT scanner or angio-suite solely for the purpose of probe placement. Rather, in case of neurological deterioration indicating the need for ptO_2_ probe placement, cranial imaging was performed anyway to rule out surgical lesions or ischemia.

### Clinical Implications and Outcome

Since ptO_2_ probes provide focal measurements reflecting oxygen tension within a small volume of brain near the sensor, the spatial relationship of the probe to the site of injury is critical when interpreting measurements [1]. Several experimental and clinical studies demonstrated lower ptO_2_ values in perilesional or abnormal brain compared to healthy brain tissue, highlighting the interaction between ptO_2_ values and the probe position [29, 32–34]. In a study of 405 patients, Ponce et al. demonstrated a stronger relationship of outcome to ptO_2_ values in abnormal brain compared to ptO_2_ values in normal-appearing brain [33]. Similarly, Lindner et al. also found a significant association between brain tissue hypoxia and poor functional outcome with ptO_2_ probes placed into perilesional brain tissue, whereas measurements in healthy tissue showed no association to outcome [34]. Kofler et al. showed that assessment of cerebral metabolism in the immediate vicinity of a brain lesion has prognostic impact [28]. However, placement of a probe into a parenchymal contusion is futile, since the ptO_2_ values of this irreversibly damaged tissue cannot be influenced by therapy [35]. Therefore, the ptO_2_ probe should be placed in potentially endangered, but salvageable perilesional brain tissue at least 1 cm away from already unviable tissue [21, 22, 28, 36]. This parallels the concept of the penumbra in ischemic stroke treatment [37].

If placed in a routine, free-hand fashion, less than half of the probes reside in perilesional tissue, while up to 17% will be within a lesion [38]. Intraoperative, frameless stereotaxy can be used to place a probe into a desired area of interest [39–41]. Unfortunately, this approach can only be applied in the operating room and depends on the availability of image-guidance by a 3D neuronavigation system. The use of CT-guidance is advantageous for several reasons: (1) Since the selection of a specific target necessitates cranial imaging to visualize the pathology and CT is used in most instances, no additional transports are necessary, (2) a postinsertional CT scan is recommended in all cases and facilitated without further transportation and (3) the technique is easy to use, safe and allows for correctional maneuvers before the probe is inserted into the brain parenchyma.

Our technique allowed us to insert ptO_2_ probes specifically into areas of critical perfusion, which are at risk for tissue hypoxia. Decreasing ptO_2_ measurements in these endangered areas lead to 59 therapeutic interventions. Thereby, prevention of ischemic tissue damage in the area of the probe was possible in all except one case. Importantly, eight therapeutic adjustments were made in that patient based on the ptO_2_ measurements, reflecting the attempt to prevent infarction with aggressive treatment. Thus, ischemic infarct was probably due to unsuccessful treatment rather than failure of ptO_2_ measurement. Furthermore, we were able to limit ischemic damage to vascular territories outside the area of the probe. This underscores the importance of correct placement of a probe into the most critically perfused area with the highest risk for ischemia.

Specific monitoring of areas at risk and aggressive treatment probably had a positive effect on outcome: eight patients (72.73%) reached an excellent outcome with a mRS 1 or 2, while only three patients (27.72%) had an unfavorable outcome with a mRS of 4. Formal comparison to other studies is difficult because of the heterogeneity of our cohort. All patients with TBI reached a good outcome, despite a maximal initial GCS of 8. In a recent, large prospective trial of patients with severe TBI and a GCS ≤ 8 only 50% reached a favorable outcome [42]. Five of seven patients (71.43%) suffering from SAH reached a good outcome in our cohort. Traditionally, vasospasm reduces the frequency of good outcome after SAH from 70 to 44% and increases mortality from 17 to 31% [43]. Our results also compare favorably with the prospective cohort of the recent HIMALAIA-trial, where 48.78% of patients had a poor outcome [44]. However, the outcome of our study group has to be interpreted cautiously, because the cohort is a mix of SAH, TBI, ICH, and other pathologies with a small sample size in a retrospective, single institution setting.

Comparison of the study group to a historical control group with conventionally placed ptO_2_ probes from our institution supports the finding that specific placement of a ptO_2_ probe in an area at risk has the potential to improve the value of ptO_2_ monitoring and ultimately outcome. This is reflected by a significantly higher rate of diagnostic and therapeutic interventions per probe per day in the study group compared to the control group. This, in turn, might have contributed to a significantly better outcome at six months in the study group.

### Radiation Exposure

The radiation exposure with an average number of scans of 3.91 per patient represents the disadvantage of our method. In order to reduce the radiation exposure, the scans were routinely limited to the supraorbital cranium. Thereby, the mean DLP was reduced to 1595.17 mGycm. Importantly, this includes a scan after the procedure in order to confirm the location of the probe and exclude hemorrhage in the vicinity of the probe, whereby the measurement would be jeopardized. A confirmatory scan is recommended in clinical practice as well as in ongoing trials (BONANZA), and imposes a radiation exposure of 600–700 mGycm by itself if performed as a regular CT scan [1, 45]. Of note, as an alternative to ptO_2_ measurements in the diagnosis of vasospasms, angio-CT combined with perfusion-CT imaging is often used and imposes a radiation exposure of 3000–3400 mGycm [46–48].

We acknowledge the need for reducing radiation exposure when CT-guided probe insertion is used clinically. In our practice, a first scan was performed after placement of the bolt, another after introduction of the probe sheath with the stylet, and another after the insertion of the probe itself. With more experience in the CT-guided technique, the scan after insertion of the probe sheath with the stylet can be omitted. This has the potential to reduce the radiation exposure by almost a quarter and will be employed in the future.

Although radiation exposure is a concern, the potential benefit of a better outcome through improved monitoring and aggressive treatment in this patient population with a critical prognosis likely offsets the harm by the radiation exposure.

### Limitations

Several limitations apply to our analysis. Our results were obtained by a retrospective analysis. Furthermore, this was a single-center study with a limited number of patients that lacks external validation. Directly comparing patient groups is of limited value due to the heterogeneity of the patient population including different severities and various pathologies. However, the heterogeneity of our cohort is also one of the strengths of our study, because the consistency of our results across various pathologies renders the results more generalizable.

We selected a historical control group with conventionally placed probes from our institution. Interpretation of the results of the comparison to our study group warrants caution for several reasons: (1) although the difference did not reach statistical significance, the study group was considerably younger than the control group, which in turn impacts outcome. (2) The higher rate of interventions in the study group could simply reflect an overall more aggressive treatment approach, irrespective of the placement of a ptO_2_ probe. (3) Duration of measurement of ptO_2_ was significantly longer in the study group, which might also reflect an overall more aggressive treatment approach. However, this could also originate from the clinician’s impression of less valuable monitoring data in the control group, which might have shortened the duration the probe was left in situ. (4) Since ptO2 probes were routinely placed by CT-guidance after October 2017 and in conventional technique before, the difference in treatment intensity and outcome might originate in an improvement in neurocritical care over time, since two separate time periods are compared. (5) Finally, the retrospective nature and small sample size of our study and control group impose the potential for selection, performance, and other biases. In order to reduce potential biases, we excluded patients who had care withdrawn within 24 h from the control group.

## Conclusions

Since ptO_2_ probe measurements only represent small brain volumes, concise probe placement is critical. CT-guidance is an accurate and easy technique for placement of a ptO_2_ probe in a particular area of interest in patients with critical cerebral oxygen supply. By applying our technique of individualized ptO_2_ probe placement, the value of monitoring probably can be improved, which has the potential to translate into better patient outcome.
